# A new species of *Oxynetra* from Mexico (Hesperiidae, Pyrginae, Pyrrhopygini)

**DOI:** 10.3897/zookeys.667.6080

**Published:** 2017-04-10

**Authors:** Andrew D. Warren, Nick V. Grishin

**Affiliations:** 1 McGuire Center for Lepidoptera and Biodiversity, Florida Museum of Natural History, University of Florida, 3215 Hull Rd., UF Cultural Plaza, PO Box 112710, Gainesville, FL, 32611-2710, USA; 2 Howard Hughes Medical Institute and Departments of Biophysics and Biochemistry, University of Texas Southwestern Medical Center, 5323 Harry Hines Blvd, Dallas, TX, 75390-9050, USA

**Keywords:** Biodiversity, mimicry, skipper butterflies, *Prunus*, Biodiversidad, mimetismo, mariposa hesperido, *Prunus*

## Abstract

*Oxynetra aureopecta* **sp. n.** is described from the Sierra Madre Oriental of east-central Mexico. Visually similar to Mesoamerican *O. hopfferi* Staudinger, 1888 in having five orange bands on the abdomen above, it is diagnosed by orange forecoxae and palpi beneath, narrower forewing hyaline bands and a prominent 6% difference in the COI DNA barcode sequence. It is the northernmost representative of the *hopfferi* species group that also includes *O. stangelandi* Grishin & Burns, 2013, characterized by a single-banded abdomen and currently known only from the Area de Conservación Guanacaste in northwestern Costa Rica. Both *O. hopfferi* and *O. stangelandi* possess white forecoxae and ventral palpi. This new discovery brings the total number of *Oxynetra* C. & R. Felder, 1862 species to five.

## Abstract (es)

*Oxynetra aureopecta* **sp. n.** se describe de la Sierra Madre Oriental, en el centro-este de México. Visualmente similar a la especie mesoamericana *O. hopfferi* Staudinger, 1888 en tener cinco bandas de color naranja en el abdomen anterior, se diagnostica por forecoxae y palpos naranja debajo, bandas hialinas más estrechas en la ala anterior, y una diferencia destacada de 6% en la secuencia de código de barras de ADN COI. Es el representante más septentrional del grupo de especies *hopfferi* que también incluye *O. stangelandi* Grishin & Burns, 2013, que se caracteriza por una banda en el abdomen y actualmente conocida solamente desde el Área de Conservación Guanacaste en el noroeste de Costa Rica. Tanto *O. hopfferi* y *O. stangelandi* poseen forecoxae y cara ventral de palpos blancos. Este nuevo descubrimiento eleva el número total de especies de *Oxynetra* C. & R. Felder a cinco.

## Introduction

Butterflies are loved for the colorful patterns of their wings. However, the lack of scales resulting in wing transparency sometimes is more appealing than colors. Most prominently known in the Clearwings (Nymphalidae: Ithomiini) and some Satyrs (Nymphalidae: Haeterini) (Warren et al. 2016), transparent wings are rarely found in Skippers (Hesperiidae). *Oxynetra* C. & R. Felder, 1862, from the Pyrrhopygini tribe, is perhaps the foremost example. These arctiid moth mimics (Grishin et al. 2013) are some of the most unusually patterned Hesperiidae, with broad hyaline discal (and sometimes subapical) bands on the wings and frequently orange-banded abdomens.

*Oxynetra* is a neotropical genus (type species *O. semihyalina* C. & R. Felder, 1862) of four species. South American *O. semihyalina* and *O. confusa* Staudinger, 1888 possess larger scale-free areas on the wings. In addition to the discal band, they have forewing subapical hyaline spots, which are larger and rounder in *O. semihyalina*. Their sexes are similar, but females have rounder wings. Mesoamerican *O. hopfferi* Staudinger, 1888, and *O. stangelandi* Grishin & Burns, 2013 lack the subapical hyalinity and their discal bands are narrower. *Oxynetra hopfferi* is characterized by its striking five-banded abdominal pattern, while *O. stangelandi* has a single complete abdominal band, as does *O. semihyalina*. The two Mesoamerican species (the *hopfferi* group) are extreme in sexual dimorphism: females lack hyalinity altogether and have black wings, sometimes with white fringes. Due to such extremism, a female of *O. hopfferi* was originally described not only as a separate species, but also in a distinct genus: *Dis annulatus* Mabille, 1889.

Unlike *O. semihyalina* and *O. confusa*, *O. hopfferi* is very rare in collections; we know only 12 male and 4 female specimens world-wide (Warren et al. 2016). *Oxynetra stangelandi* is currently known only from the type series of 10 specimens (4 males and 6 females) reared from Area de Conservación Guanacaste (ACG) in northwestern Costa Rica. Its caterpillars feed on *Prunus annularis* Koehne, adding a new family, Rosaceae, to those eaten by Pyrrhopygini in ACG (Grishin et al. 2013). The *hopfferi* group is comprised of cloud forest species recorded from above 1000 m in elevation. Its unique wasp-like appearance creates the potential for cryptic species to be overlooked within *O. hopfferi*.

## Materials and methods

Specimens were examined from the following collections: Los Angeles County Museum of Natural History, Los Angeles, CA, USA (**LACM**); National Museum of Natural History, Smithsonian Institution, Washington, DC, USA (**USNM**); Colección Nacional de Insectos “Dr. Alfredo Barrera Marin”, Museo de Historia Natural y Cultura Ambiental de la Ciudad de México, Mexico (**CNIABM**); McGuire Center for Lepidoptera and Biodiversity, Florida Museum of Natural History, University of Florida, Gainesville, FL, USA (**MGCL**); Natural History Museum, London, UK (**BMNH**); Museum für Naturkunde, Berlin, Germany (**ZMHB**); Senckenberg Museum für Tierkunde, Dresden, Germany (**MTD**); O. H. H. Mielke, Curitiba, Paraná, Brazil, together with the collection of Departamento de Zoologia, Universidade Federal do Paraná, Curitiba, Brazil (**OM-DZUP**) and Instituto Nacional de Biodiversidad, Santo Domingo de Heredia, Costa Rica (**INBio**). Wing venation terminology follows Steinhauser (1981). Length measurements are in metric units and were made from photographs of specimens taken next to a scale and magnified on a computer screen. Photographs of specimens were taken with Nikon E5000 and Nikon D200, D800 cameras through a Nikkor 105 mm f/2.8G AF-S VR Micro lens. Images were assembled and edited in Photoshop CS5.1.

Legs, crumbs and pieces of muscle tissue from the thorax of dissected specimens (plucked from the abdomen attachment site), or a distal part of an abdomen (dropped into lysis buffer, and after overnight incubation at 56°C transferred into 10% KOH for genitalia dissection) were used to extract genomic DNA with the Macherey-Nagel (MN) NucleoSpin tissue kit following the manufacturer's protocol. The lysis buffer volume was scaled down to 70 μl for legs and volumes of subsequent reagents were proportionally reduced. Genomic DNA was eluted in a total volume of 40–100 μl MN BE buffer (concentration of DNA as measured by Promega QuantiFluor® dsDNA System was from near 0 to 20 ng/μl, mostly around 1 ng/μl, depending on specimen age and storage conditions) and was stored at -20°C. PCR was performed using Invitrogen AmpliTaq Gold 360 master mix in a 20 μl total volume containing less than 1 ng of template DNA (usually 0.5–1 μl of DNA extract) and 0.5 μM of each primer. The following pairs of primers were used: sCOIF (forward, 5’-ATTCAACCAATCATAAAGATATTGG-3’) – Meg-mCOIR (reverse, 5’-CCAGTWCCTGYACCATTTTCTAC-3’), and Ven-mCOIF (forward, 5’-GCATTCCCTCGTATAAATAATA-3’) – sCOIR (reverse, 5’-TAAACTTCTGGATGTCCAAAAAATCA-3’), to amplify the barcode in two overlapping segments. The PCR reaction was cleaned by enzymatic digestion for the whole barcode amplifications, ID tag amplification, and sequences amplified in more than two segments, with 4 μl Shrimp Alkaline Phosphatase (20 U/μl) and 1 ul Exonuclease I (1 U/μl) from New England Biolabs. For sequences obtained in two segments, due to the frequent presence of primer dimers and other short non-specific PCR products, Agencourt Ampure XP beads or Invitrogen E-Gel EX Agarose Gels (followed by Zymo gel DNA recovery kit) were used to select the DNA products of expected length. Sequences were obtained with primers used in PCR. Sanger sequencing was performed with Applied Biosystems Big Dye Terminator 3.1 kit on ABI capillary instrument in the DNA Sequencing Core Facility of the McDermott Center at UT Southwestern. The resulting sequence traces were proofread in FinchTV (http://www.geospiza.com/Products/finchtv.shtml). Sequences and accompanying specimen data were submitted to GenBank and received accession numbers KT272397 and KT272398.

Additional DNA sequences were downloaded from GenBank (https://www.ncbi.nlm.nih.gov/genbank) or BOLD (http://www.boldsystems.org). Many of these sequences have been reported in Janzen et al. (2011) and photos of specimens are available from the Area de Conservación Guanacaste (ACG) online database (Janzen and Hallwachs 2014) and BOLD database (Ratnasingham and Hebert 2007) to confirm or suggest identifications. Sequences were aligned manually since they matched throughout their length without insertions or deletions. The Phylogeny.fr server (http://www.phylogeny.fr) was used with the Hamming distance model (Dereeper et al. 2008) to compute evolutionary distances from aligned DNA sequences and dendrograms were built with BioNJ (Gascuel 1997). Maximum Likelihood analysis was performed using RAxML (version 7.0.4) under several substitution models, such as GTRCAT, GTRGAMMA, and GTRGAMMAI (Stamatakis 2006). Rapid RAxML bootstrap values (-x option, and “-f a” for complete analysis) were computed to judge the confidence of tree nodes. Bayesian Inference was performed with MrBayes v3.2.1 (Huelsenbeck and Ronquist 2001, Ronquist and Huelsenbeck 2003). Models with 1, 2 and 6 states were used (nst = 1, 2, 6), with optimized fraction of invariant positions (propinv), gamma distribution parameter (gamma) or both (invgamma). The COI alignment was treated as a single partition, or analyzed as three partitions by codon position. Generations were carried out until convergence (standard deviation of split frequencies less than 0.01) and the first 25% were discarded as “burn in.” Posterior probabilities of nodes computed by MrBayes were used as the indicators of confidence.

## Results

Working in the Colección Nacional de Insectos, “Dr. Alfredo Barrera Marin” (CNIABM), ADW found and photographed a damaged (missing two wings and distal ends of antennae) *Oxynetra* specimen from the R. Mϋller collection, the only *Oxynetra* specimen known from Mexico (Veracruz: Presidio). It was assumed that it is probably *O. hopfferi* near its northern distribution limits. Upon further analysis, differences from typical Costa Rican and Panamanian *O. hopfferi* were noticed, including the orange “chest” and palpi below, narrower forewing hyaline band and the lack of a ventral hindwing postdiscal white spot in cell CuA_2_-2A. A second specimen from Mexico, very similar to the Mϋller specimen and collected 300 kilometers to the northwest (Hidalgo: Puerto Caballo), identified as “*Oxynetra hopfferi*,” surfaced when NVG was browsing the Hesperiidae collection of the Los Angeles County Museum of Natural History (LACM). The morphological differences from *O. hopfferi* were consistent with the Mϋller specimen, and the DNA COI sequence of the LACM specimen revealed a remarkable 6% difference (about 40 different base pairs) from *O. hopfferi* and 4.7% (31 base pairs) from *O. stangelandi*. Despite the five-banded abdomen and the presence of a white streak on the dorsal hindwing posteriad of vein 2A shared with *O. hopfferi*, but not with O. *stangelandi*, it appears that the Mexican *Oxynetra* might be more distant from both *O. hopfferi* or *O. stangelandi*, a sister-species pair differing by about 3% (about 20 base pairs) in the COI barcode (Grishin et al. 2013). We therefore describe the Mexican taxon as a distinct species.

### Oxynetra aureopecta

Taxon classificationAnimaliaLepidopteraHesperiidae

A. Warren & Grishin sp. n.

http://zoobank.org/9325E7C1-E690-4E38-9709-BC83613B4D18

Figs 1–4 , 11 part

#### Description.

**Figures 1–10.**
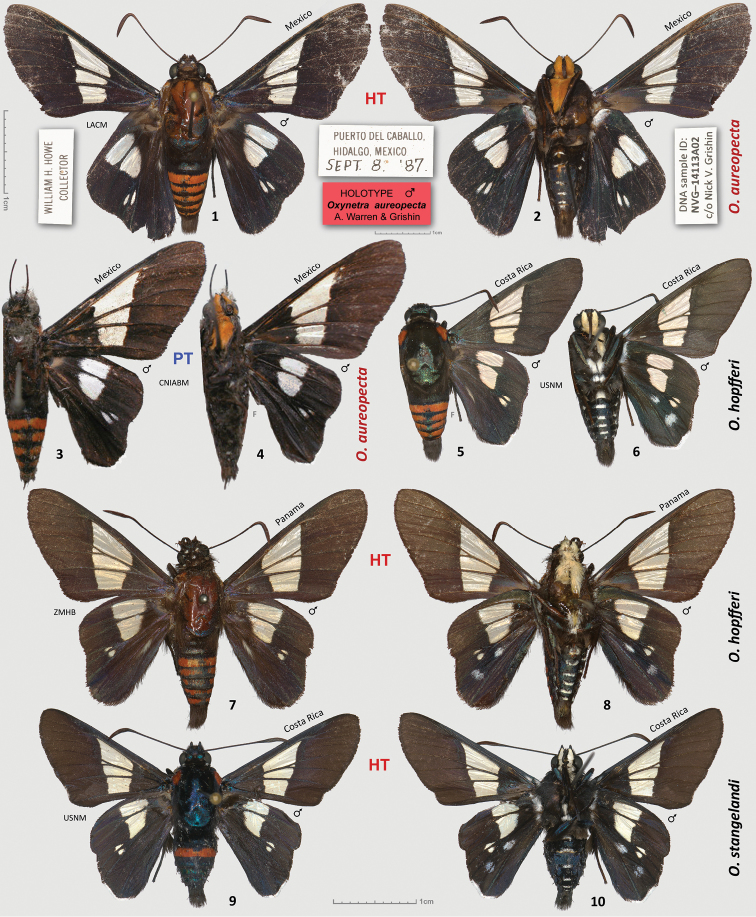
Males of *Oxynetra*. **1**–**4** *O. aureopecta* sp. n. holotype (**1**–**2**) and paratype (**3**–**4**), data in text **5**–**6** *O. hopfferi*, Costa Rica: Puntarenas, Monteverde, 1997, voucher 97-ZFuentes-055 [USNM] **7**–**8** *O. hopfferi* holotype, Panama: Chiriqui [ZMHB] **9**–**10** *O. stangelandi* holotype, Costa Rica: Guanacaste, eclosed on 19.VIII.2002, voucher 02-SRNP-23284 [USNM]. Dorsal and ventral surfaces are shown on odd- and even-numbered figures, respectively. Labels are shown for the holotype of the new species and are reduced 1.5 times compared to specimens: smaller scale bar above the top labels refers to labels, and larger scale bars refer to specimens. Pinholes and some imperfections have been removed to emphasize actual wing patterns.

Male (Figs 1–4): right forewing length 21.8 mm (holotype). Hindwing (HW) narrow and elongate; forewing (FW) extending well beyond it. Outer wing margin slightly concave at cell CuA_2_‒1A+2A of FW and at cells between veins M_1_ and M_3_ of HW. Dorsal and ventral FW (including fringe) brownish-black with blue-purple metallic sheen, and with a hyaline band from anterior edge of discal cell (where it is widest) to vein 1A+2A; band divided into three aligned parts by dark-scaled veins CuA_1_ and CuA_2_; its outer edge does not extend beyond the origin of vein M_3_; the hyaline wedge at the very base of cell M_3_‒CuA_1_ is either very small (holotype) or lacking (paratype). Dorsal HW concolorous with FW (except fringe around tornus white), with two median, large, aligned, hyaline spots in cell Sc+R1‒Rs (oval) and in discal cell (roughly triangular), separated by vein Rs, and suggesting continuation of FW band; smaller, postmedian pair of hyaline streak-like spots in proximal ends of cells M_3_‒CuA_1_ and CuA_1_‒CuA_2_; a small patch of white hair-like scales in the discal part of cell 2A-3A. Ventral HW similar to dorsal, but with wing base white and with a diffuse patch of white scales in discal part of cell CuA2‒1A+2A. Antenna black; nudum (missing on paratype) medium brown, 20 segments. Head and body primarily brownish-black with a blue-purple sheen, marked as follows: two small white spots at base of antenna and one small spot at dorsoposterior margin of eye; first and second segments of palpi orange ventrally; forecoxae orange; white patches at posterior margin of each sternite and on sides of abdomen; large orange spot on anterior half of tegula; five orange bands across terga III to VII. Genitalia not dissected. Female unknown.

#### Barcode sequence of the holotype.

Genbank Accession KT272397, voucher NVG-14113A02, 658 base pairs:

AACTTTATATTTTATATTTGGAATTTGAGCAGGAATAATTGGAACTTCATTAAGATTACTAATTCGAACTGAATTAGGTA

CCCCCGGATCTTTAATTGGAAATGATCAAATTTACAATACTATCGTAACAGCTCATGCATTTATTATAATTTTTTTTATA

GTTATACCTATTATAATTGGAGGATTTGGAAATTGATTAATTCCTTTAATATTAGGAGCACCAGATATAGCTTTTCCTCG

TATAAATAATATAAGATTTTGATTATTACCCCCATCTTTAACTCTTTTAATTTCAAGAAGAACTGTAGAAAATGGTGTTG

GAACTGGATGAACAGTTTATCCCCCCCTCTCTTCTAATATTGCTCATCAAGGGGCCTCAGTTGATTTAGCTATTTTTTCT

CTTCATTTAGCAGGAATTTCTTCAATTTTAGGAGCTATTAATTTTATTACAACAATTATTAATATACGAATTAAAAATTT

ATCTTTTGATCAAATACCTCTTTTTGTATGAGCAGTAGGAATTACTGCATTACTATTATTATTATCTTTACCTGTATTAG

CAGGTGCTATTACTATACTTTTAACAGATCGAAATATTAATACTTCTTTTTTTGACCCAGCAGGTGGAGGAGATCCTATT

TTATATCAACATTTATTT

#### Types.

Holotype ♂ (Figs 1–2) with the following four rectangular labels: white, printed and handprinted - || PUERTO DEL CABALLO, | HIDALGO, MEXICO | SEPT. 8. ‘87. ||; white, printed - || WILLIAM H. HOWE | COLLECTOR ||; red, printed - || HOLOTYPE ♂ | *Oxynetra aureopecta* | A. Warren & Grishin ||; white printed - || DNA sample ID: | NVG-14113A02 | c/o Nick V. Grishin ||. The holotype is in the Los Angeles County Museum of Natural History, Los Angeles, CA, USA (LACM). Paratype ♂ (Figs 3–4) from MEXICO: Veracruz, Presidio, R. Mϋller Coll., in CNIABM.

#### Type locality.

MEXICO: Hidalgo: Puerto del Caballo, elevation about 1020 m, GPS approximately 21°10', −98°55'.

#### Etymology.

The name of this new species refers to its orange “chest”, including palpi beneath and forecoxae, which is the most obvious diagnostic character. The name is an adjective.

#### Distribution and habitat.

*Oxynetra aureopecta* is known only from the holotype and one paratype, both males, from Puerto del Caballo, Hidalgo, and Presidio, Veracruz, which are about 300 km from each other in eastern Mexico. Puerto del Caballo is situated at about 1020 m in the central Sierra Madre Oriental, along Hwy. 85, about 4.5 air km southwest of the San Luis Potosí border. This area is comprised of cloud forest vegetation, near the transition at lower elevations to tropical deciduous forest. The Presidio, Veracruz area has been extensively modified, and very few forested areas remain; material labeled from Presidio includes species typical of tropical deciduous and cloud forest habitats. The similar *O. hopfferi* and *O. stangelandi* are both cloud forest denizens, the latter reported to use *Prunus annularis* (Rosaceae) as a larval foodplant (Grishin et al. 2013). Various *Prunus* species are likely present in the Puerto del Caballo area, including *P. samydoides* Schlecht., *P. salicifolia* HBK. and *P. microphylla* (Kunth) Hemsl. (Standley 1920, Pennington and Sarukhán 2005), which could serve as foodplants for *O. aureopecta*.

#### Diagnosis.

**Figure 11.**
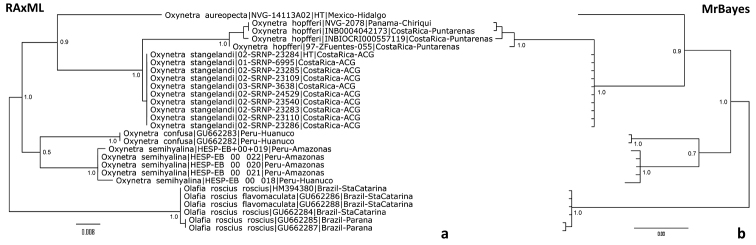
COI DNA barcode trees of *Oxynetra* species. The trees are obtained by **a** RAxML under “GTRGAMMA” model; and **b** MrBayes under “propinv” model with 2 states (see Materials and Methods) and show identical topology. The taxa are arranged in the same sequence in both trees. The trees are rooted with *Olafia* Nemésio, 2005 sequences. Bootstrap fractions (a) and posterior probabilities (b) are shown (except for nodes within species). Sequences with NVG- voucher codes were obtained in this work. For other sequences, ACG voucher codes (with -SRNP- and -ZFuentes-, Janzen & Hallwachs 2014), INBio voucher codes (starting with INB, Grishin et al. 2013), GenBank accessions (starting from GU and HM, http://genbank.gov/), or Ernst Brockmann collection voucher codes (with HESP-EB) are indicated for each sequence. The general locality of each specimen is indicated.

This new species belongs to *Oxynetra* because it has the traits of the genus as defined by Evans (1951). In particular, “F end cell upright, convergent with termen at tornus” (Evans, 1951). By the COI DNA barcode, this species groups within *Oxynetra* as a sister to the *O. hopfferi* and *O. stangelandi* clade, in accord with similarities in appearance to these two species, and away from the *O. semihyalina* and *O. confusa* clade (Fig. 11). A combination of the following characters identifies males of *O. aureopecta*: (1) orange “chest”, i.e., forecoxae and palpi beneath (males of both *O. hopfferi* and *O. stangelandi* have white forecoxae and palpi); (2) no postdiscal white spot on ventral hindwing in cell CuA_2_-2A (the other two species possess this white spot in addition to the discal spot in that cell); (3) narrower forewing hyaline band barely extending distad the base of M_3_-CuA_1_ cell, and very small (or lacking) spot at the base of this cell (the band prominently extends distad of M_3_-CuA_1_ cell and the hyaline spot at the base of this cell is more prominent in the other two species); (4) longer (and narrower), streak-like spots in hindwing cells M_3_-CuA_1_ and CuA_1_-CuA_2_ (the spots, in particular the one in cell CuA_1_-CuA_2_, are rounder in the other two species); (5) five orange bands on the abdomen above, similarly to *O. hopfferi* (only a single complete band is present in *O. stangelandi*, Fig. 9); (6) a weakly developed white streak of a few hair-like scales near the anal fold on dorsal hindwing (the streak is absent in *O. stangelandi*, Fig. 9, but is well-defined in *O. hopfferi*, Figs 5, 7); **(7)** DNA COI barcode 6.1% and 4.7% different from that of *O. hopfferi* and *O. stangelandi*, respectively. Characters (1) and (3) appear to be the most easily observed. The female of *O. aureopecta* is unknown but may be mostly black similar to females of the other two species.

#### Discussion.

The description of *O. aureopecta* adds a fifth species to *Oxynetra*, and confirms the occurrence of the genus in Mexico. While the damaged paratype specimen in CNIABM has been examined by many researchers, its authenticity has been questioned since it was apparently the only specimen of the genus labeled from Mexico (De la Maza et al. 1991, Warren 2000). Thus, the discovery of the holotype specimen in the LACM, in much better condition than the paratype—and nearly identical in appearance—confirms the provenance of *Oxynetra* in Mexico. Based on the known distribution of *O. aureopecta* in cloud forest habitats of the Sierra Madre Oriental, we suspect that the species might be endemic to Mexico.

However, *Oxynetra* species remain unknown from Guatemala, El Salvador, Honduras and Nicaragua. Given the rarity of species in the *hopfferi* group- the only group of the genus thus far known to occur in Mesoamerica, it may be that the genus has merely gone undetected in those countries (significant rearing efforts were necessary to detect the presence of *O. stangelandi* in northwestern Costa Rica). Therefore, much more fieldwork must be conducted before the overall distributions of *Oxynetra* species in Mesoamerica can be defined.

## Supplementary Material

XML Treatment for Oxynetra aureopecta
